# Treatment delay in childhood pleural tuberculosis and associated factors

**DOI:** 10.1186/s12879-020-05496-4

**Published:** 2020-10-27

**Authors:** Huai-Zheng Gong, Chao Han, Feng-Lian Yang, Chun-Fang Wang, Jun-Li Wang, Mao-Shui Wang

**Affiliations:** 1 Department of Lab Medicine, Shandong Provincial Chest Hospital, Cheeloo College of Medicine, Shandong University, Jinan, China; 2 Department of Geriatrics, Shandong Mental Health Center, Jinan, China; 3 School of Pharmacy, Youjiang Medical University for Nationalities, Baise, China; 4 Department of Lab Medicine, The Affiliated Hospital of Youjiang Medical University for Nationalities, Baise, China

**Keywords:** Children, Pleural tuberculosis, Treatment, Delay, Risk factor

## Abstract

**Background:**

Delay in diagnosis and treatment worsens the disease and clinical outcomes, which further enhances the transmission of tuberculosis (TB) in the community. Therefore, this study aims to assess treatment delay and its associated factors among childhood pleural TB patients in China.

**Methods:**

Between January 2006 and December 2019, consecutive patients aged ≤15 years with definite or possible pleural TB were included for analysis. Treatment delay duration was defined as the time interval from the onset of symptoms to treatment initiation and was stratified into two categories: < 30 days, ≥30 days (median delay day is 30 days). The electronic medical records of children were reviewed to obtain demographic characteristics, clinical characteristics, laboratory examinations, and radiographic findings. Univariate and multivariate logistic regressions were used to explore the factors associated with treatment delay in patients.

**Results:**

A total of 154 children with pleural TB were included, with a mean age of 12.4 ± 3.3 years. The median treatment delay was 30 days (interquartile range, 10–60 days) and 51.3% (*n* = 79) of patients underwent a treatment delay. Multivariate analysis revealed that heart rate (≤92 beats/min, age-adjusted OR = 2.503, 95% CI: 1.215, 5.155) and coefficient of variation of red cell distribution width (RDW-CV, ≥12.9%, age-adjusted OR = 4.705, 95% CI: 2.048, 10.811) were significant risk factors for treatment delays in childhood pleural TB.

**Conclusion:**

Our findings suggested that a significant treatment delay occurs among children with pleural TB in China. Patients with a low heart rate or a high RDW-CV experienced delays in the initiation of anti-TB therapy. Therefore, well awareness of the associations between clinical characteristics and treatment delay may improve the management of children with pleural TB and enable us to develop preventive strategies to reduce the treatment delay.

**Supplementary information:**

**Supplementary information** accompanies this paper at 10.1186/s12879-020-05496-4.

## Background

According to the World Health Organization (WHO), 10.0 million people fell ill with tuberculosis (TB), 1.2 million TB deaths occurred among HIV-negative people and 251,000 TB deaths occurred among HIV-positive people in 2018 [1]. In children, although Bacille Calmette–Guérin (BCG) vaccination confers protection, children still account for 11% of total TB cases [1]. Pleural TB is thought to be a common form of TB disease in children, with an approximate rate of 4–15% [2–4]; however, in paediatric patients, pleural TB can complicate 36–73% of cases of pulmonary TB [5–8]. Although several descriptive cases highlighting the clinical characteristics of childhood pleural TB have been reported [5, 6, 9–11], the epidemiological characteristics, such as surgical risk and delay in treatment, remain unclear. Therefore, further analysis is required to improve strategies for the optimal management of childhood pleural TB.

Although improved health policies and novel assays facilitate TB diagnosis confirmation and quick treatment initiation, delays in the treatment of pulmonary TB remain common. Longer intervals from the onset of TB symptoms to the initiation of anti-TB therapy may lead to a worse outcome and an increased transmission risk [12]. For example, in a meta-analysis, treatment delay was identified as a predictor of poor treatment outcome (death and failure) [13]. A study conducted by Bajehson et al. found a significant negative association between treatment delay and survival [14]. One possible explanation for this is that delay is significantly associated with clinical severity at presentation [15]. In addition, in a study conducted by Cheng S et al., delayed TB treatment was associated with a significantly increased risk of pulmonary sputum smear positivity and pulmonary cavity [16]. Hence, avoiding treatment delay may decrease the risk of transmission. Moreover, a previous report suggested that delay in anti-TB treatment is an important risk factor for chronic obstructive pulmonary disease [17].

Treatment delay in pleural TB in adulthood was also previously investigated, and several risk factors were identified [18]. In addition, although delays in the management of childhood TB have been characterized [19–21], the situation of delayed treatment in childhood TB with pleural involvement is less clear. Therefore, this retrospective study aimed to assess the characteristics of childhood pleural TB and estimated the risk factors for delayed treatment initiation.

## Methods

This study protocol was approved by the Ethics Committee of Shandong Provincial Chest Hospital (SPCH). Written informed consent was waived because of the retrospective design of the study and the anonymous nature of the data collection.

Between January 2006 and December 2019, consecutive patients aged ≤15 years and admitted to SPCH with suspected childhood pleural TB were included for potential analysis. Definite pleural TB was confirmed by mycobacterial culture (sputum, pleural effusion, or pleural tissues) or suggested by pathological evidence (such as caseous necrosis or Langhans’ giant cells). Patients with a positive TB assay result (such as TB RT-PCR, acid-fast bacilli (AFB) smear, or both) and clinical symptoms (or a positive response to anti-TB therapy) were diagnosed with possible pleural TB.

Treatment delay duration was defined as the time interval from the onset of symptoms (by patient or family recall) to treatment initiation and was stratified into two categories: < 30 days and ≥ 30 days (median delay day is 30 days). The electronic medical records of children with definite and possible pleural TB were reviewed to obtain demographic characteristics, clinical characteristics, laboratory examinations, and radiographic findings.

All analyses were performed using SPSS version 16.0 (SPSS, Chicago, IL, USA). The data are presented as the mean ± SD. In univariate analysis, differences between groups were tested for significance with the Mann-Whitney *U* test or *t* test for continuous variables and the χ^2^ test or Fisher exact test for categorical variables. The associations between the parameters were analysed using the Spearman correlation test. In addition, univariate logistic regression analysis was used to estimate risk factors for treatment delay, and variables with a *P* value < 0.1 were included in the multivariate logistic regression model. Prior to multivariate logistic regression analysis, continuous variables were transformed into categorical variables by receiver operating characteristic curve (ROC) analysis, and the cut-off value was chosen as the point corresponding to the maximal sum of the sensitivity and specificity. Then, multivariate logistic regression analysis was performed, the corresponding odds ratios (OR) and 95% confidence interval (CI), adjusted by age, were calculated, and statistical significance was indicated when a two-sided *P* value was < 0.05 [18]. Prior to multivariate logistic regression analysis, continuous variables were transformed into categorical variables by receiver operating characteristic curve (ROC) analysis, and the cut-off value was chosen as the point corresponding to the maximal sum of the sensitivity and specificity. The Hosmer-Lemeshow goodness-of-fit test was used to test the accuracy of the model. A *P* value < 0.05 was considered significant for the difference estimated.

## Results

### Patient characteristics

The median treatment delay was 30 days (interquartile range, 10–60 days). Table 1 and Supplementary Table 1 show the demographic data, clinical characteristics, laboratory examinations, and radiographic findings of children with pleural TB. A total of 154 children with pleural TB (definite, 123 cases; possible, 31 cases) were included in our study. The mean age was 12.4 ± 3.3 years. Boys accounted for 64.9% (100 patients), and 103 (100%) were HIV-negative. The mean weight was 46.1 ± 16.2 kg. Among the 154 children, 93 (60.4%) were from rural areas. The vital signs were as follows: blood pressure, 111.4 ± 12.3/69.2 ± 8.5 mmHg; heart rate, 97.6 ± 16.0 beats/min; respiratory rate, 22.5 ± 2.7 breaths/min; and temperature, 37.2 ± 0.9 °C.

**Table 1:** Univariate analysis of the demographic data associated with treatment delay in childhood pleural TB

	Total (n)	Below the Median (< 30 days, n)	Above the Median (≥30 days, n)	*P* value^*^	OR (95% CI)
N	154	75 (48.7%)	79 (51.3%)		
Treatment delays (median, interquartile range; days)	30 (10–60)	10 (9–20)	60 (30–120)	0.963	
Vital Signs					
Temperature (°C)	37.2 ± 0.9	37.4 ± 0.9	37.1 ± 0.9	0.018	0.642 (0.445, 0.925)
Heart rate (beats/min)	97.6 ± 16.0	100.7 ± 15.2	94.7 ± 16.2	0.021	0.975 (0.955, 0.996)
Respiratory rate (breaths/min)	22.5 ± 2.7	22.9 ± 2.5	22.2 ± 2.9	0.075	0.896 (0.794, 1.011)
Systolic pressure (mmHg)	111.4 ± 12.3	110.8 ± 13.5	112.0 ± 11.1	0.525	
Diastolic pressure (mmHg)	69.2 ± 8.5	69.4 ± 8.8	69.0 ± 8.4	0.766	
Medical history					
Contact history of TB	20 (13.0%)	10 (13.3%)	10 (12.7%)	0.901	
Transferred times	2.1 ± 1.0	2.0 ± 0.8	2.2 ± 1.2	0.313	
Transferred from a teaching hospital	91 (59.1%)	44 (58.7%)	47 (59.5%)	0.917	
Times of hospitalization	2.0 ± 1.6	1.9 ± 1.6	2.0 ± 1.6	0.679	
Surgical treatment	29 (18.8%)	10 (13.3%)	19 (24.1%)	0.093	2.058 (0.887, 4.779)
Symptoms					
Cough	84 (54.5%)	44 (58.7%)	40 (50.6%)	0.317	
Fever	134 (87.0%)	69 (92.0%)	65 (82.3%)	0.08	0.404 (0.146, 1.114)
Chest pain	69 (44.8%)	35 (46.7%)	34 (43.0%)	0.651	
Dyspnea	42 (27.3%)	22 (29.3%)	20 (25.3%)	0.576	
Sputum production	29 (18.8%)	11 (14.7%)	18 (22.8%)	0.201	
Complications					
Cavity	7 (4.5%)	3 (4.0%)	4 (5.1%)	0.752	
Loculated effusion	27 (17.5%)	11 (14.7%)	16 (20.3%)	0.364	
Empyema	27 (17.5%)	7 (9.3%)	20 (25.3%)	0.012	3.293 (1.301, 8.335)
Blood analysis					
White blood cell (10^9^/L)	7.3 ± 2.6	7.3 ± 2.4	7.2 ± 2.7	0.776	
Red blood cell (10^12^/L)	4.4 ± 0.5	4.4 ± 0.5	4.5 ± 0.5	0.071	1.908 (0.947, 3.846)
Hemoglobin (g/L)	121 ± 14	121 ± 12	122 ± 16	0.595	
Hematocrit	36.3 ± 3.9	36.2 ± 3.5	36.9 ± 4.3	0.297	
Mean corpuscular volume (fL)	82.4 ± 5.1	83.1 ± 4.9	81.8 ± 5.2	0.137	
Mean corpuscular haemoglobin (pg)	27.3 ± 2.1	27.7 ± 2.2	27.0 ± 2.0	0.045	0.846 (0.719, 0.997)
Mean corpuscular haemoglobin concentration (g/L)	331 ± 13	333 ± 13	330 ± 12	0.102	
Platelet (10^9^/L)	353 ± 129	366 ± 123	340 ± 134	0.225	
Neutrophil (10^9^/L)	5.1 ± 6.2	5.5 ± 8.5	4.7 ± 2.2	0.454	
Lymphocyte (10^9^/L)	1.7 ± 0.9	1.8 ± 1.1	1.7 ± 0.7	0.503	
Monocyte (10^9^/L)	0.8 ± 0.4	0.8 ± 0.4	0.7 ± 0.4	0.829	
Coefficient of variation of red cell distribution width (%)	13.9 ± 1.8	13.3 ± 1.1	14.4 ± 2.2	0.001	1.609 (1.223, 2.116)
Erythrocyte sedimentation rate (mm/h)	40.8 ± 26.4	46.5 ± 23.4	35.2 ± 28.1	0.011	0.983 (0.970, 0.996)

*TB* Tuberculosis, *OR* Odds ratio, *CI* Confidence interval. ^*^P values were obtained from the univariate logistic regression analysis

Among the enrolled children, 20 (13.0%) had a TB contact history, and 29 (18.8%) were treated with surgical procedures. The mean number of transferred times between hospitals was 2.1 ± 1.0, and the mean number of hospitalizations was 2.0 ± 1.6. Most of the children (91, 59.1%) were transferred from a teaching hospital. The symptoms were as follows: fever (134, 87.0%), cough (84, 54.5%), chest pain (69, 44.8%), dyspnoea (42, 27.3%), and sputum production (29, 18.8%). The radiographic findings showed that 7 patients (4.5%) had cavities, 27 (17.5%) had loculated effusion, 62 (40.3%) had effusion on the left side, 74 (48.1%) on the right side and 18 (11.7%) on both sides. Out of the 154 study patients, 27 (17.5%) had empyema, 89 (57.8%) had pulmonary TB, 3 (1.9%) had bronchial TB, 13 (8.4%) had tuberculous lymphadenitis, 4 (2.6%) had tuberculous meningitis, and 7 (4.5%) had milliary TB.

Other characteristics, such as clinical chemistry analysis (serum or pleural effusion), blood cell analysis, and flow cytometry analysis, are also shown in Table 1 and Supplementary Table 1.

### Comparisons between groups with or without treatment delay

For the comparison of continuous variables between groups (below vs above the median (30 days)), Mann-Whitney *U* tests were used, and the statistical analysis showed that differences in temperature (*P* < 0.05), heart rate (*P* < 0.05), mean corpuscular haemoglobin (MCH, *P* < 0.05), coefficient of variation of red cell distribution width (RDW-CV, *P* < 0.01), and erythrocyte sedimentation rate (ESR, *P* < 0.05) were significant when comparing the two groups. The differences in other continuous variables did not reach significance (all *P* > 0.05). For dichotomous variables, chi-square analysis was performed, and the analysis suggested that the difference between the two groups was significant in empyema (*P* < 0.05). The differences in other continuous variables did not reach significance (all *P* > 0.05).

### Univariate and multivariate analysis

Univariate analysis was performed to estimate each risk factor for the delay in the treatment of childhood pleural TB. Temperature (OR = 0.642, 95% CI: 0.445, 0.925), heart rate (OR = 0.975, 95% CI: 0.955, 0.996), MCH (OR = 0.846, 95% CI: 0.719, 0.997), RDW-CV (OR = 1.609, 95% CI: 1.223, 2.116), ESR (OR = 0.983, 95% CI: 0.970, 0.996) and empyema (OR = 3.293, 95% CI: 1.301, 8.335) were associated with treatment delays in childhood pleural TB (all *P* < 0.05).

To make the results as readily understandable as possible, continuous variables were converted into dichotomous categorical variables based on the cut-off points determined with ROC analysis, and the corresponding optimal cut-off values were 37.2 °C, 92 beats/min, 27.2 pg, ≥12.9% and 25 mm/h for temperature, heart rate, MCH, RDW-CV, and ESR, respectively. Further multivariate analysis (Hosmer–Lemeshow goodness-of-fit test: χ^2^ = 5.978, df = 8, *P* = 0.650) revealed that heart rate (≤92 beats/min, age-adjusted OR = 2.503, 95% CI: 1.215, 5.155) and RDW-CV (≥12.9%, age-adjusted OR = 4.705, 95% CI: 2.048, 10.811) were significant risk factors for treatment delays in childhood pleural TB (Table 2).

**Table 2:** Age-adjusted OR for risk factors associated with treatment delay in childhood pleural TB

	Age-adjusted OR	*P* value
Heart rate (≤92 beats/min)	2.503 (1.215, 5.155)	0.013
Coefficient of variation of red cell distribution width (≥12.9%)	4.705 (2.048, 10.811)	< 0.001

*TB* Tuberculosis, *OR* Odds ratio, *CI* Confidence interval

Further analysis showed that heat rate had a significant association with cough and fever (all *P* < 0.05), a positive correlation with respiratory rate (r = 0.669, *P* < 0.001), temperature (r = 0.347, *P* < 0.001), ESR (r = 0.336, *P* < 0.001), platelets (r = 0.218, *P* = 0.008), and lactate dehydrogenase (serum, r = 0.191, *P* < 0.001), and a negative correlation with weight (r = − 0.247, *P* = 0.002), age (r = − 0.236, *P* = 0.003), haemoglobin (r = − 0.214, *P* = 0.009), haematocrit (r = − 0.207, *P* = 0.012), systolic pressure (r = − 0.199, *P* = 0.015), and red blood cell count (r = − 0.165, *P* = 0.046). RDW-CV had a significant association with effusion sites, chest pain, and tuberculous meningitis (all *P* < 0.05), a positive correlation with CD4+/CD8+ (r = 0.503, *P* < 0.001), CD19+ (r = 0.282, *P* = 0.019), and lactate dehydrogenase (serum, r = 0.203, *P* = 0.032), and a negative correlation with mean corpuscular haemoglobin concentration (r = − 0.575, *P* < 0.001), MCH (r = − 0.521, *P* < 0.001), mean corpuscular volume (r = − 0.341, *P* < 0.001), CD3+ (r = − 0.265, *P* = 0.023), haemoglobin (r = − 0.247, *P* = 0.003), glucose (serum, r = − 0.215, *P* = 0.014), albumin (serum, r = − 0.179, *P* = 0.032), and weight (r = − 0.176, *P* = 0.036).

## Discussion

Treatment delays in TB patients are a major problem in most countries with a high TB burden. In our cohort, a median delay of 30 days from symptom onset to treatment initiation was found in cases of childhood pleural TB. Our study suggests that a lower heart rate or a higher RDW-CV were associated with treatment delay. Remarkably, the identification of these characteristics will help to improve the management of children with pleural TB. Moreover, TB control can be facilitated through early initiation of effective treatment.

Until now, delay in the treatment of TB has been reported worldwide and remains a threat to TB control [12, 22–26]. In a previous study, although some data on delay in seeking treatment among pleural TB patients were obtained [18], the reason for this delay in paediatric patients remains unclear. In our study, a 30-day median treatment delay was found in childhood pleural TB patients, which was in line with the findings of pulmonary TB patients [27–30]. In addition, a similar median delay (30 days) was reported in pleural TB patients irrespective of age [18]; however, the median treatment delay varied widely between studies. One explanation for this difference is the definition of treatment delay. For example, in a study in Taiwan, treatment delay, which was defined as the period between sputum collection and the start of treatment, was reported in pulmonary TB patients with a median period of 120 days [31]. In another study, treatment delay defined from the registration date at Regional TB Centre to treatment initiation was reported at a median delay of 13 days. Treatment delay was also considered as the period between diagnosis confirmation and treatment initiation [32]. In our study, approximately two-thirds of patients included for the analysis visited at least two different hospitals before admission to our hospital. In fact, collaboration between medical providers is poor, and unnecessary diagnoses significantly delayed the initiation of anti-TB treatment [33]. In addition, although approximately 60% of paediatric patients were admitted to a teaching hospital, most remained undiagnosed. Hence, health system delays could be considered a main factor influencing the treatment delay in the treatment of childhood pleural TB in China.

In univariate analysis, several characteristics, such as temperature, empyema, MCH, and ESR, were identified as risk factors associated with treatment delay in children with pleural TB (all *P* < 0.05); however, due to the *P* values, these characteristics were not included in the final multiple logistic models. Previously, the absence of fever was significantly associated with total delay in patients with pulmonary TB, pleural TB, tuberculous meningitis, and endobronchial TB [18, 34–37]. This might be because fever is usually considered a serious symptom, and less delay was found in seeking health services [35]; however, no significant difference was found between the two groups. Fever, as an initiation symptom, is recalled by the patient before admission to the hospital, And the temperature is measured upon admission. Therefore, disease progression and previous treatment may have a significant impact and lead to a different result. Empyema was also a reported risk factor for treatment delay in pleural TB of adults. This could be explained by inappropriate treatment being responsible for the development of tuberculous empyema in most patients [38, 39]. The MCH level is considered an important parameter for anaemia diagnosis, and decreased MCH indicates anaemia caused by iron deficiency. In addition, Malbasa M et al. reported that anaemia was significantly associated with delayed treatment of pulmonary TB [37]. In our study, anaemia was not associated with delayed treatment of childhood pleural TB; however, in view of the association between MCH and anaemia, anaemia remains one of the most important causes of treatment delay for childhood pleural TB. Elevated ESR levels may aid in the diagnosis of childhood pleural TB, and the corresponding treatment delay was reduced significantly [40].

In multiple logistic analysis, heart rate and RDW-CV, which we believe were not previously described in the literature, were identified as risk factors for the delayed treatment of childhood pleural TB. In fact, further analysis showed that heart rate and RDW-CV have significant associations with some known risk factors for treatment delay for TB disease, which have been previously characterized, such as fever and anaemia [18, 37]. Interestingly, tachycardia has been reported as a risk factor for treatment delay [37]. This finding is similar to the results of our study. Although the criteria of tachycardia may be of some benefit in the identification of patients, it may also be overly restrictive. Therefore, a heart rate cut-off value chosen based on a rational analysis may lead to better management of children with pleural TB with a high risk of delayed treatment. In fact, treatment delay caused by the two clinical factors should be classified as a health care delay. If greater attention is given to the association between the clinical manifestations and treatment delay, the delay in treatment in children with pleural TB may be decreased. In addition, except for AFB smears, most TB assays (such as Xpert and culture) have not been conducted at general hospitals in China. Hence, our findings may be generalized to primary care centres because there is no requirement of special machines.

Although some interesting findings have been obtained, several limitations of our study must be remarked. First, due to the retrospective design, selection bias was a significant concern in our study. Second, a recall bias may be present because patients or their guardians may fail to remember the exact time of symptom onset. Third, the study was a single centre observation cohort study, and patients were only recruited from our hospital. Thus, some findings may not be generalized to other districts in China. Fourth, because of a significant proportion of possible TB, the diagnostic delay was hard to define and not evaluated in our study. Furthermore, the power to detect statistical significance between the examined associations was limited due to the relatively small sample size. The results of this study should be interpreted with some caution.

## Conclusions

In conclusion, our study suggested that a significant treatment delay occurs among children with pleural TB in China. Patients with a low heart rate or a high RDW-CV experience delays in the initiation of anti-TB therapy. Hence, increased awareness of the associations between clinical characteristics and treatment delay may improve the management of children with pleural TB and achieve a better outcome.

## Supplementary information

**Additional file 1: Table S1.** Univariate analysis of the demographic data associated with treatment delay in childhood pleural TB.

## Data Availability

The data analyzed in this study can be accessed by sending a request to the corresponding author.

## Abbreviations

TB: Tuberculosis
BCG: Bacilli Calmette–Guérin
AFB: Acid-fast bacilli
ROC: Receiver operating characteristic curve
OR: Odds ratios
95% CI: 95% confidence interval
MCH: Mean corpuscular hemoglobin
RDW-CV: Coefficient of variation of red cell distribution width
ESR: Erythrocyte sedimentation rate
